# Worldwide SARS-CoV-2 haplotype distribution in early pandemic

**DOI:** 10.1371/journal.pone.0263705

**Published:** 2022-02-16

**Authors:** Andrea Cairo, Marilena V. Iorio, Silvia Spena, Elda Tagliabue, Flora Peyvandi

**Affiliations:** 1 Angelo Bianchi Bonomi Hemophilia and Thrombosis Center and Fondazione Luigi Villa, Fondazione IRCCS Ca’ Granda Ospedale Maggiore Policlinico, Milan, Italy; 2 Research Department, Fondazione IRCCS Istituto Nazionale dei Tumori, Milan, Italy; 3 Department of Pathophysiology and Transplantation, Università Degli Studi di Milano, Milan, Italy; Centers for Disease Control and Prevention, UNITED STATES

## Abstract

The world is experiencing one of the most severe viral outbreaks in the last few years, the pandemic infection by SARS-CoV-2, the causative agent of COVID-19 disease. As of December 10^th^ 2021, the virus has spread worldwide, with a total number of more than 267 million of confirmed cases (four times more in the last year), and more than 5 million deaths. A great effort has been undertaken to molecularly characterize the virus, track the spreading of different variants across the globe with the aim to understand the potential effects in terms of transmission capability and different fatality rates. Here we focus on the genomic diversity and distribution of the virus in the early stages of the pandemic, to better characterize the origin of COVID-19 and to define the geographical and temporal evolution of genetic clades. By performing a comparative analysis of 75401 SARS-CoV-2 reported sequences (as of December 2020), using as reference the first viral sequence reported in Wuhan in December 2019, we described the existence of 26538 genetic variants, the most frequent clustering into four major clades characterized by a specific geographical distribution. Notably, we found the most frequent variant, the previously reported missense p.Asp614Gly in the S protein, as a single mutation in only three patients, whereas in the large majority of cases it occurs in concomitance with three other variants, suggesting a high linkage and that this variant alone might not provide a significant selective advantage to the virus. Moreover, we evaluated the presence and the distribution in our dataset of the mutations characterizing the so called “british variant”, identified at the beginning of 2021, and observed that 9 out of 17 are present only in few sequences, but never in linkage with each other, suggesting a synergistic effect in this new viral strain. In summary, this is a large-scale analysis of SARS-CoV-2 deposited sequences, with a particular focus on the geographical and temporal evolution of genetic clades in the early phase of COVID-19 pandemic.

## Introduction

In nineteen months after the declaration of the SARS-CoV-2 pandemic [1], the scientific community has been struggling to understand the complexity of this novel coronavirus of still debated zoonotic origin [2], the clinical symptoms [3, 4], the risk factors, the potential treatments, in the urgent effort to contain the infection, to predict potentially serious disease outcomes, to find a cure. In 2021, several SARS-CoV-2 vaccines have been generated and approved, and several other are still in clinical trials [5]. The circulation of SARS-CoV-2 before the pandemic declaration has been ascertained, and several efforts have been made to track the worldwide spreading and the genetic changes that originated different viral strains.

The first case of COVID-19 was reported in Wuhan, China, in December 2019 and, despite the relatively low mortality (approximately 2% on average worldwide, as of December 10^th^ 2021, *https*:*//ourworldindata*.*org/mortality-risk-covid#the-case-fatality-rate*) and the high percentage of asymptomatic or pauci-symptomatic subjects (over 80%), the viral outbreak has literally caused a dramatic collapse of the health care system in the most hit countries, as in Italy, where the mortality rate reached over 14% between May and August 2020.

In the urgent race to find efficient drugs and decrease the complications, it has been immediately clear that a deeper understanding of the genomic diversity of this virus was crucial. Indeed, the existence of different strains and their temporal and geographical distribution could provide relevant information on: how the virus spread all over the world, the possible acquisition of selective advantages, the most conserved sequences suitable for a vaccine design.

Since the first complete genomic sequence of SARS-CoV-2 release on January 5^th^ 2020 [1, 2], thousands of additional sequences have been deposited. Different virus strains have been reported [6], and the accumulation of recurrent variants [7, 8]. Here, we evaluated the occurrence of different SARS-COV-2 clades and their geographical distribution starting from 75401 sequences deposited in two major available databases, GISAID and NCBI, from the beginning of COVID-19 pandemic to September 2020.

## Material and methods

### Data acquisition

The VCF file containing all identified variants in 77648 SARS-CoV-2 genomes and relative information about geographical area, date of sampling and genome length were downloaded on September 29^th^ 2020 from http://covseq.baidu.com/. Viral sequences were collected from major available databases: GISAID (74303) and NCBI (3345). Genomes without detailed date of sampling, geographical localization and with a sequence shorter than 29000 nucleotides were excluded. After filtering, 75401 viral genomes were included in the analysis. The list of all analyzed sequences with the collection dates is available in **S1 Table.**

### Sequence and mutational analysis

The reference sequence used for our analysis is NC_045512.2 (NCBI RefSeq). Multiallelic variants were splitted using Bcftools [9]. Variants were annotated using SnpEff v.4.5covid19 [10]. Data manipulation and descriptive analysis were performed using python package PANDAS [11]. Viral genomes and relative variants were organized in a binary matrix. The identification of clades and haplotypes was performed including in the analysis only the variants identified in at least 1000 viral sequences. We created a sub-dataset containing the 53 variants identified as present in more than 1000 viral sequences (**S1 Table)** and different haplotypes were identified and counted using groupby PANDA function (**S2 Table**). The sequences were classified into clades and deriving haplotypes based on the mutational distribution. Frequency of each variant in all countries was calculated (**S3 Table**). and represented by clustering analysis and relative heatmap, which were performed with R 4.0.0s. Only countries with at least 50 viral sequences were included in the clustering analysis.

## Results and discussion

The geographical distribution of available sequences is reported in **S1 Fig.** Almost 58% of all available sequences was obtained from European centers, principally United Kingdom, followed by North America (~20%), Oceania (~11%) and Asia (~8%). The samples were collected from December 2019 to August 17^th^ 2020 at different times across different countries, and analyzing their temporal distribution it is worth noting that few sequences were early obtained (January 2020) in United States (January 19^th^ 2020), Australia (January 22^nd^ 2020) and North Europe (January 23^rd^ 2020) (**S2 Fig**).

China represents the country with the highest percentage of unmutated viral genomes (3%) (**Table 1**).

**Table 1:** SARS-CoV-2 sequence distribution.

Continent/Country	N° Sequences	N° Seq. with 0 variant (%)
**EUROPE**	43508	81(0.1)
Austria	425	1 (-)
Denimark	742	1 (-)
Germany	392	1 (-)
Iceland	581	5 (0.8)
Italy	215	0 (-)
Sweden	526	1 (-)
Switzerland	708	2 (-)
UK	32867	69 (0.2)
**NORTH AMERICA**	15272	27 (0.2)
Usa	14580	26 (0.2)
Canada	553	1 (-)
**SUD AMERICA**	1327	1 (-)
Brazil	838	1 (-)
**AFRICA**	984	2 (-)
South Africa	231	1 (-)
**ASIA**	6363	78 (1.2)
China	1093	32 (3.0)
India	2413	2 (-)
**OCEANIA**	7947	1 (-)
Australia	7688	1 (-)

**Fig 1:**
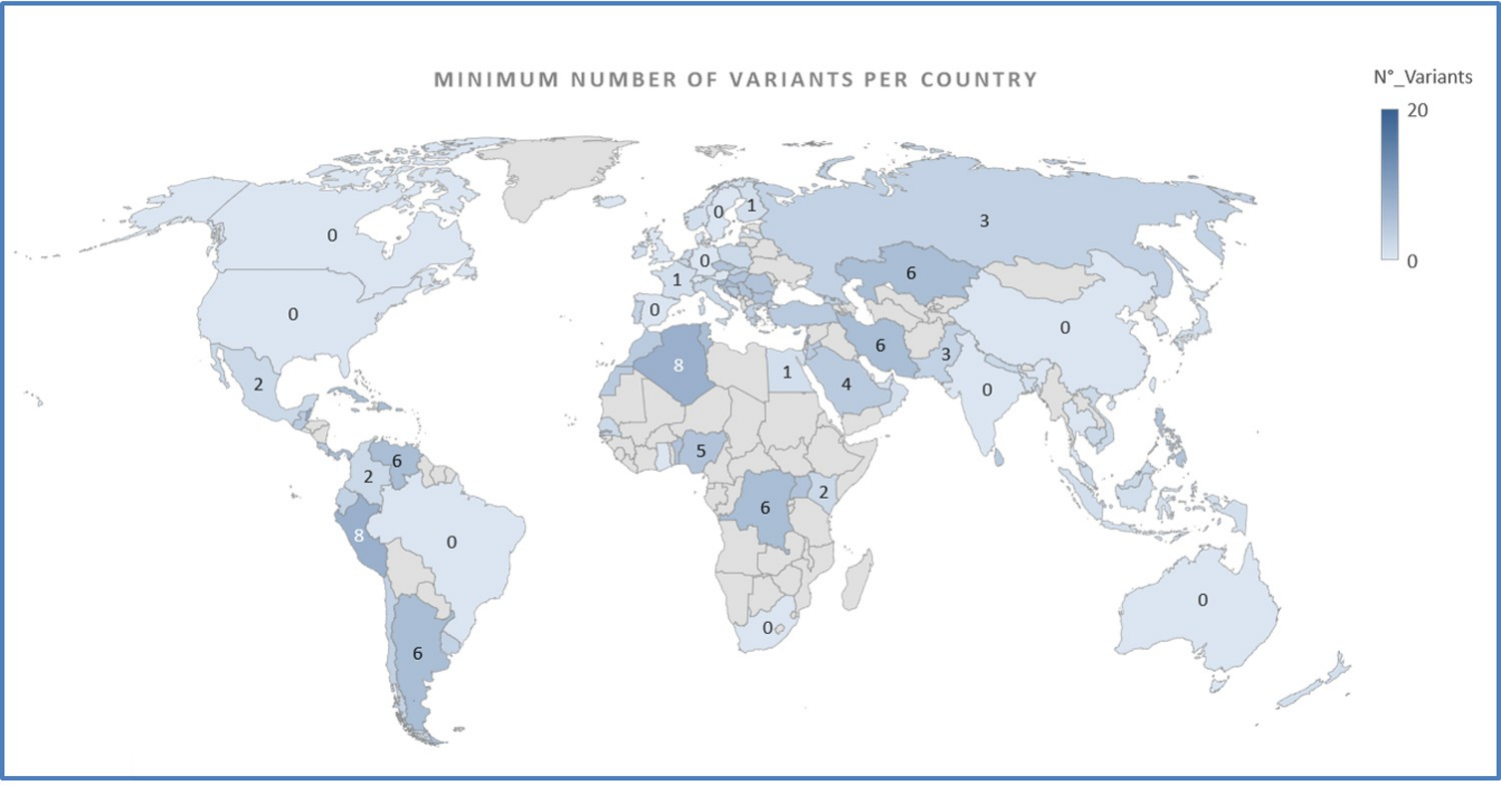
Geographical distribution of the minimum number of variants per sequence.

Observing the distribution of viral unmutated sequences, it could be noticed how USA, Northern Europe, Australia, South Africa, Brazil, Canada and India have been directly involved in the spread of the pandemic after China (**Fig 1**). Analyzing the minimum number of variants per sequence in each country we could indeed track the mutational evolution and the relative geographical distribution, and the circulation timing of the virus. Importantly, this approach allowed us to hypothesize that some countries severely hit by COVID-19, as Italy and France, were characterized by the spread of already mutated forms of the virus (**Fig 1**).

A total of 26539 variants were identified (**S4 Table**): 57% missense, 28% synonymous, 7% insertions/deletions, 2% stop variants, 7% of variants being localized in the untranslated regions, 4% in the 3’UTR and 3% in the 5’UTR **(S3A Fig).** The number of variants per sequence spans mainly from 0 to 20; the majority of sequences carried from 7 to 9 variants and only few cases (431) had more than 20 variants (**S3B Fig**). A plausible hypothesis could be that, whereas few variants may have provided such favorable features as an improved infectivity, a higher number of variants did not result in a selective advantage. Variants present in at least 1000 sequences are reported in **Table 2** (N = 53). As expected, nonsense mutations were not found among the most frequent variants.

**Table 2:** Most frequent SARS-CoV-2 variants. Only the variants identified in at least 1000 viral sequences are reported.

Genomic Position*	Gene	c.DNA	Protein	Type of variant	Date of first observation	Country	N°of sequences	% of sequences	Functional data
23403	S	c.1841A>G	p.Asp614Gly	missense	24/01/2020	China	57820	77%	Increased infectivity (13;15;16)
14408	ORF1ab	c.14144C>T	p.Pro4715Leu	missense	24/01/2020	China	57609	76%	
3037	ORF1ab	c.2772C>T	p.Phe924Phe	synonymous	24/01/2020	China	57562	76%	
241	ORF1ab	c.-25C>T	/	5’UTR	24/01/2020	China	56487	75%	
28881	N	c.608G>A	p.Arg203Lys	missense	27/01/2020	Australia	25974	34%	
28883	N	c.610G>C	p.Gly204Arg	missense	27/01/2020	Australia	25927	34%	
28882	N	c.609G>A	p.Arg203Arg	synonymous	27/01/2020	Australia	25923	34%	
25563	ORF3a	c.171G>T	p.Gln57His	missense	16/02/2020	Singapore	16214	22%	
29836	/	c.4453_4519del	/	3’UTR	20/01/2020	China	14181	19%	
1059	ORF1ab	c.794C>T	p.Thr265Ile	missense	16/02/2020	Singapore	12589	17%	
11083	ORF1ab	c.10818G>T	p.Leu3606Phe	missense	17/01/2020	China	7821	10%	
14805	ORF1ab	c.14541C>T	p.Tyr4847Tyr	synonymous	09/02/2020	United Kingdom	6401	8%	
28144	ORF8	c.251T>C	p.Leu84Ser	missense	26/12/2019	China	5481	7%	
8782	ORF1ab	c.8517C>T	p.Ser2839Ser	synonymous	26/12/2019	China	5347	7%	
26144	ORF3a	c.752G>T	p.Gly251Val	missense	21/01/2020	China	5228	7%	
1163	ORF1ab	c.898A>T	p.Ile300Phe	missense	27/01/2020	Australia	4180	6%	
18555	ORF1ab	c.18291C>T	p.Asp6097Asp	synonymous	27/01/2020	Australia	3940	5%	
16647	ORF1ab	c.16383G>T	p.Thr5461Thr	synonymous	27/01/2020	Australia	3827	5%	
23401	S	c.1839G>A	p.Gln613Gln	synonymous	27/01/2020	Australia	3821	5%	
7540	ORF1ab	c.7275T>C	p.Thr2425Thr	synonymous	27/01/2020	Australia	3800	5%	
22992	S	c.1430G>A	p.Ser477Asn	missense	27/01/2020	Australia	3770	5%	Increased infectivity (21, 22)
20268	ORF1ab	c.20004A>G	p.Leu6668Leu	synonymous	25/01/2020	Australia	3433	5%	
23731	S	c.2169C>T	p.Thr723Thr	synonymous	02/03/2020	Denmark	3319	4%	
10097	ORF1ab	c.9832G>A	p.Gly3278Ser	missense	02/03/2020	Denmark	3308	4%	
18060	ORF1ab	c.17796C>T	p.Leu5932Leu	synonymous	19/01/2020	USA	2596	3%	
17858	ORF1ab	c.17594A>G	p.Tyr5865Cys	missense	21/02/2020	USA	2542	3%	
17747	ORF1ab	c.17483C>T	p.Pro5828Leu	missense	21/02/2020	USA	2477	3%	
2558	ORF1ab	c.2293C>T	p.Pro765Ser	missense	09/02/2020	United Kingdom	2250	3%	
2480	ORF1ab	c.2215A>G	p.Ile739Val	missense	09/02/2020	United Kingdom	2107	3%	
18877	ORF1ab	c.18613C>T	p.Leu6205Leu	synonymous	21/02/2020	Lebanon	2014	3%	
17247	ORF1ab	c.16983T>C	p.Arg5661Arg	synonymous	25/02/2020	United Kingdom	1672	2%	
27964	ORF8	c.71C>T	p.Ser24Leu	missense	03/03/2020	USA	1666	2%	
15324	ORF1ab	c.15060C>T	p.Asn5020Asn	synonymous	22/01/2020	China	1656	2%	
1604	ORF1ab	c.1341_1343delTGA	p.Asp448del	ins/del	05/02/2020	United Kingdom	1653	2%	
25429	ORF3a	c.37G>T	p.Val13Leu	missense	02/03/2020	USA	1629	2%	
28854	N	c.581C>T	p.Ser194Leu	missense	16/01/2020	China	1618	2%	
28580	N	c.307G>T	p.Asp103Tyr	missense	02/03/2020	Chile	1604	2%	
28311	N	c.38C>T	p.Pro13Leu	missense	26/02/2020	South Korea	1548	2%	
29829	/	c.4446_4519del	/	3’UTR	27/02/2020	Netherlands	1380	2%	
313	ORF1ab	c.48C>T	p.Leu16Leu	synonymous	27/02/2020	Canada	1369	2%	
28657	N	c.384C>T	p.Asp128Asp	synonymous	25/02/2020	France	1355	2%	
29864	/	c.4481_4517del	/	3’UTR	18/01/2020	China	1329	2%	
1440	ORF1ab	c.1175G>A	p.Gly392Asp	missense	23/02/2020	United Kingdom	1317	2%	
28863	N	c.590C>T	p.Ser197Leu	missense	25/02/2020	France	1309	2%	
9477	ORF1ab	c.9212T>A	p.Phe3071Tyr	missense	01/02/2020	China	1307	2%	
13730	ORF1ab	c.13466C>T	p.Ala4489Val	missense	14/02/2020	Singapore	1303	2%	
25979	ORF3a	c.587G>T	p.Gly196Val	missense	25/02/2020	France	1292	2%	
19839	ORF1ab	c.19575T>C	p.Asn6525Asn	synonymous	26/02/2020	Switzerland	1218	2%	
2891	ORF1ab	c.2626G>A	p.Ala876Thr	missense	23/02/2020	United Kingdom	1202	2%	
2416	ORF1ab	c.2151C>T	p.Tyr717Tyr	synonymous	29/02/2020	France	1160	2%	
29851	/	c.4468_4519del	/	3’UTR	25/01/2020	Australia	1154	2%	
6312	ORF1ab	c.6047C>A	p.Thr2016Lys	missense	04/03/2020	Taiwan	1126	1%	
23929	S	c.2367C>T	p.Tyr789Tyr	synonymous	04/03/2020	Taiwan	1063	1%	

* Numbering refers to the reference sequence: NC_045512.2.

Tracing the mutational evolution of the viral genome has been crucial to evaluate potential functional consequences and to obtain an efficient vaccine, which should preferentially target more evolutionary conserved regions. Our analysis underlines that, despite the high number of variants, the regions coding for the polyprotein ORF1ab, the spike (S) and the membrane (M) proteins are the most conserved (**S3C Fig**). This is not surprising, since most evolutionary conserved regions usually encode essential proteins. However, we confirmed the missense variant p.Asp614Gly in S protein as the most frequent (found in 57820 sequences, 77% of the total) (**Table 2)**, as described in previous studies [7, 8, 12, 13]. This variant has been shown to improve the binding affinity of the S protein to the human ACE2 receptor, reported as main entry site of the virus into human cells [14], through the cleavage of the S1/S2 domain [15]. Indeed, it was previously shown that SARS-CoV infection can be enhanced by exogenous proteases.

It has been demonstrated that the p.Asp614Gly variant confers higher infectivity [16], competitive fitness and improved transmission in human primary cells and animal models [17]. However, the variant does not seem to be associated with an increased disease severity [16], despite what was initially inferred from the correlation of mortality rate and prevalence of p.Asp614Gly in different countries [8, 18].

Given its pitoval role in receptor binding, membrane fusion and cell infectivity, the capability to induce neutralizing antibodies and T cell-immune responses and the relative low variability so far reported, the S protein has been considered a valid target for the development of SARS-CoV-2 vaccines [19–21].

It is relevant to notice that, focusing on the 53 most frequent variants, we found the p.Asp614Gly variant as a single mutation in three patients only, suggesting that this variant alone does not provide a significant selective advantage to the virus. Indeed, in the large majority of cases p.Asp614Gly occurs concomitantly with other variants, in particular with three variants, all characterized by similar frequency and located in the *ORF1ab* gene: the missense p.Pro4715Leu, the synonymous p.Phe924Phe and the c.-25C>T in the 5’ untranslated region. These four variants are indeed in strong linkage and constitute the most common clade (clade 1). We identified 4 different clades and evaluated their temporal occurrence, geographical distribution and potential connections (**Table 3).**

**Table 3:** Most frequent SARS-CoV-2 clades: Frequency and distribution.

Clade	Genomic position of variants	Date of first sample collection	Country of first detection	Africa (%)	Asia (%)	Europe (%)	North America (%)	Oceania (%)	South America (%)	All (%)
**Clade_1**	23403/14408/3037/241	25-Jan-2020	Australia	77.00	49.00	78.00	73.00	73.00	77.00	74.00
**Sub_Clade_1_A**	23403/14408/3037/241/28881/28882/28883	27-Jan-2020	Australia	19.00	23.00	41.00	5.00	52.00	50.00	33.00
**Sub_Clade_1_B**	23403/14408/3037/241/25563/1059	16-Feb-2020	Singapore	4.00	6.00	7.00	51.00	8.00	5.00	16.00
**Clade_2**	28144/8782	26-Dec-2019	China	5.00	10.00	3.00	17.00	10.00	5.00	7.00
**Clade_3**	11083/14805/26144	9-Feb-2020	UK	0.90	1.70	9.30	1.60	3.60	1.50	6.20
**Clade_4**	1440	23-Feb-2020	UK	0.10	0.20	2.80	0.10	0.60	0.10	1.70

The variants characterizing clade 1 have been identified in 55582 sequences, 74% of the total. This clade, first reported in UK in January 2020, has literally spread worldwide and particularly to European countries, where it represents 78% of sequences in (1059 cases), to North and South America (73 and 77%), Africa (77%) and Oceania (73%) (**S4 Fig).** Interestingly, this core is present in approximately 93% (201 of 215) of Italian cases.

**Fig 2:**
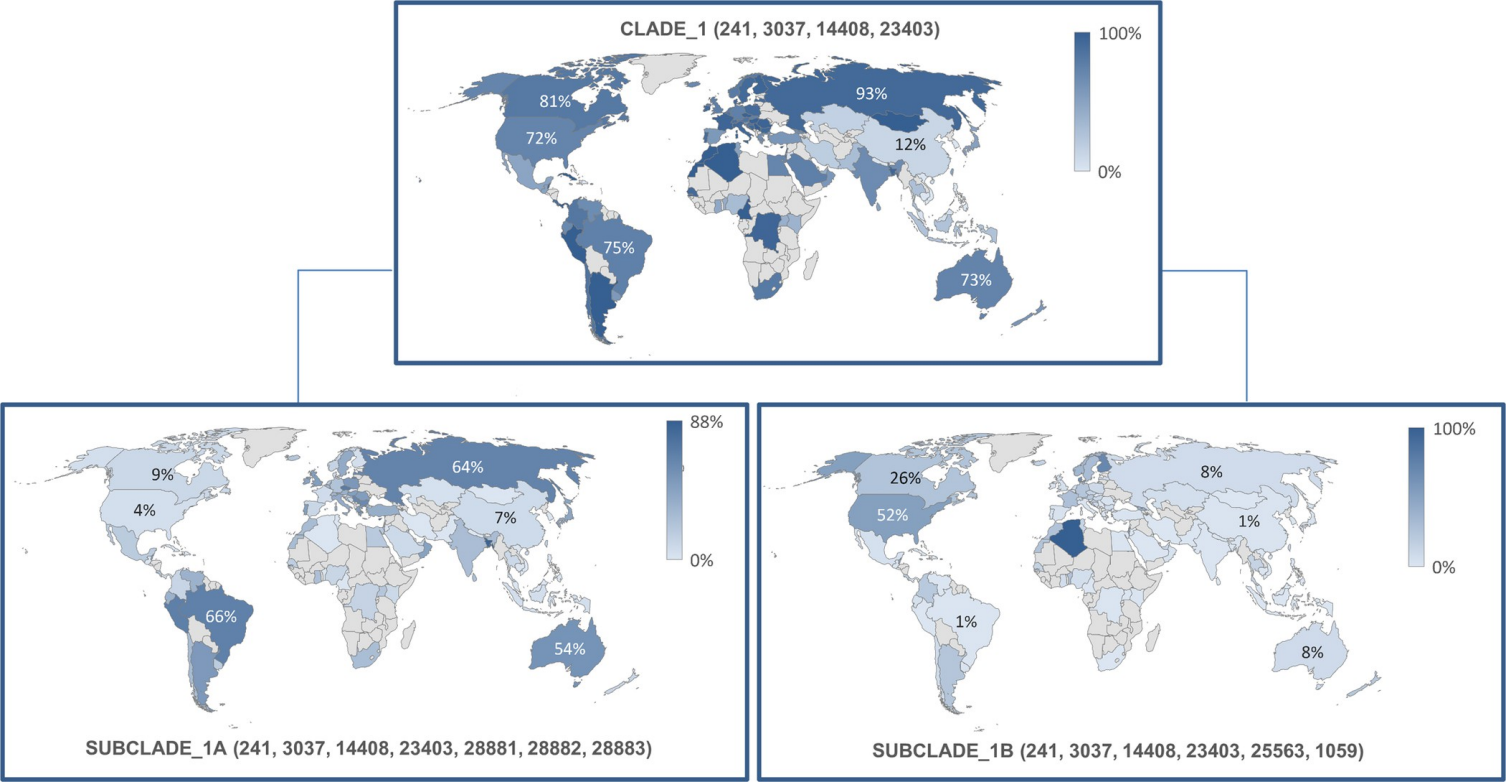
Frequency and geographical distribution of sub-clades 1A and B.

Clade 1 has likely generated two subclades, subclade_1A and subclade_ 1B, characterized by a different geographical frequency (**Fig 2**), with the presence of subclade_1A mainly in Europe (41%), Oceania (52%) and South America (50%), whereas subclade_1B is present mainly in North America (51%) (**Table 3**).

**Fig 3:**
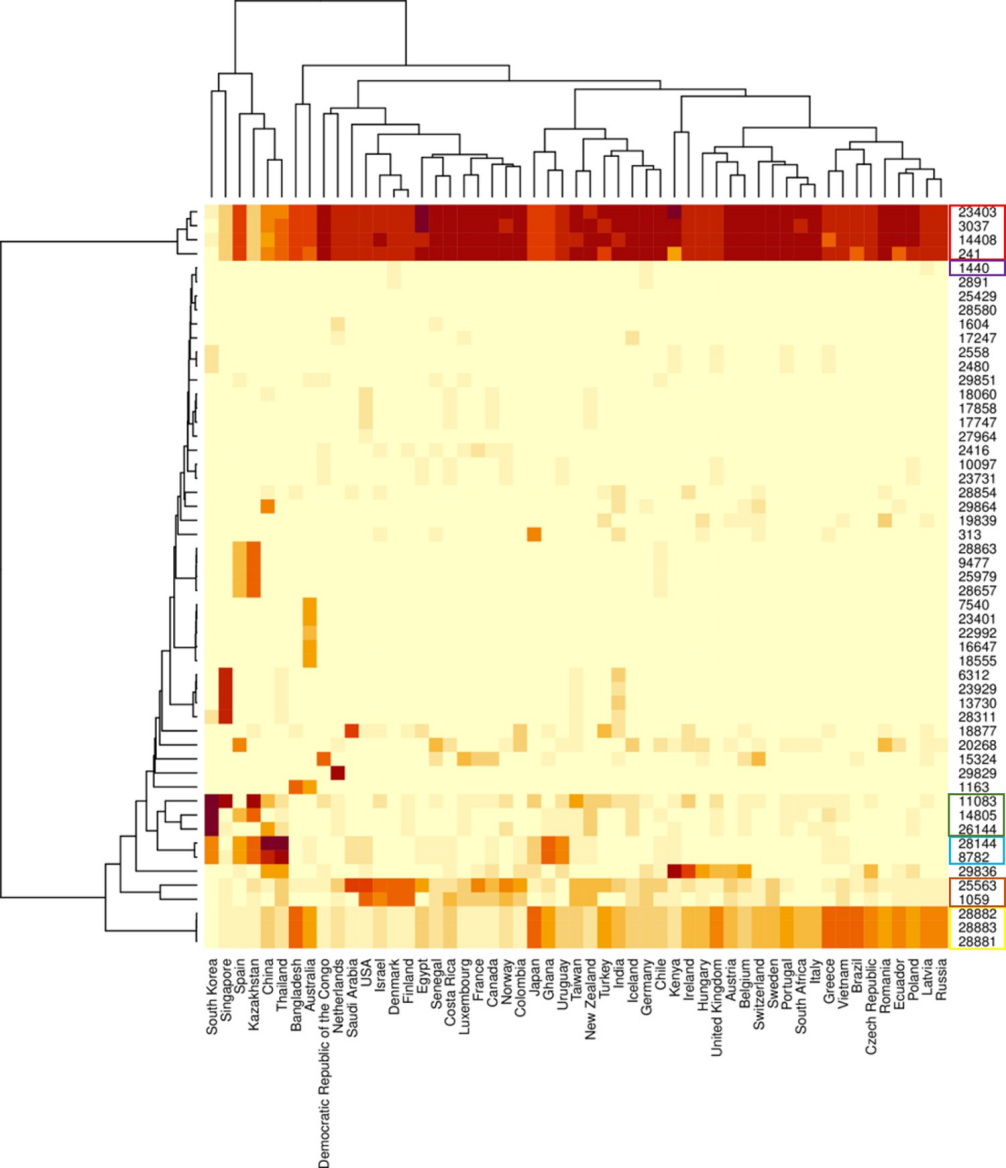
Hierarchical cluster analysis of most frequent SARS-CoV-2 variants and geographical distribution. Only countries with at least 50 viral sequences were included in the clustering analysis.

A visual representation of the spreading of different variants and relative clades is clearly inferable from the hierarchical cluster analysis reported in **Fig 3**. The plot highlights the co-presence of the four most common variants constituting clade 1 and their worldwide spreading. Additionally, it is possible to observe how the distribution of the three variants characterizing subclade_1A (28881, 28882, 28883) and the two variants peculiar of subclade_1B (25563, 1059) present an almost mutually exclusive geographical localization.

Clade 2, characterized by two different variants (8782 and 28144), is present in North America, some European countries such as Spain, some regions of Asia (China, Kazakistan and Thailand), Africa (Ghana and Nigeria) and Australia (**Fig 3** and **S5 Fig**).

Clade 3 (11083, 14805, 26144) and clade 4 (1440) appear to be more common in Europe. Despite the worldwide distribution of clade 1 variants, it is remarkable the peculiar case of South Korea, characterized by an almost exclusive presence of clade 3, and at lower frequency of clade 2 (**Fig 3**). In addition, some regions are characterized by a high frequency of different variants not related to the four major clades. 58% of sequences of Singapore presents a specific pattern of 5 variants (11083, 28311, 13730, 23929, 6312); Spain and Kazakistan curiously present the diffusion of similar virus strains characterized by the presence of 4 variants (28863, 9477, 25979, 28657) observed in 28% and 35% of the sequences, respectively. Likewise, 47% of the Australian sequences are characterized by the presence of 6 variants (7540, 23401, 22992, 16647, 18555, 1163) (**Fig 3**). The 22992 missense variant, leading to a S477A aminoacidic change, has been associated to a stronger transmission capacity [22] and higher infectivity, perhaps due to an improved binding of the S protein RBD (receptor-binding domain) to the ACE receptor [23]. Other sporadic mutations are related to a specific geographical area, as the variant 313 identified in 37% of Japanese sequences, the variants 18877 and 29829 observed in the 69% of sequences from Saudi Arabia and 90% of the sequences from Netherlands, respectively. These four identified clades constitute the core of 1213 different haplotypes, of which 548 are unique, whereas the remaining occurred in more than one patient. We report the 20 most frequent haplotypes according to the number of cases (**Table 4**).

**Table 4:** Most frequent SARS-CoV-2 haplotypes: Frequency and distribution. In bold the variants charactering Clade 1, in italics Clade 2, underlined Clade 3 and double underlined Clade 4.

Haplotype ID	Genomic position of variants	Date of first sample collection	Country	N° of sequences	% of sequences
1	**23403/14408/3037/241/**28881/28882/28883	16-Feb-2020	UK	10337	14%
2	**23403/14408/3037/241/**25563/1059	16-Feb-2020	Singapore	8511	11%
3	**23403/14408/3037/241**	3-Feb-2020	UK	5944	8%
4	**23403/14408/3037/241**/28881/28882/28883/29836	27-Feb-2020	Spain	3944	5%
5	**23403/14408/3037/241**/28881/28882/28883/1163/18555/16647/23401/7540/22992	19-Mar-2020	Australia	2900	4%
6	***28144/8782*/**18060/17858/17747	21-Feb-2020	USA	2242	3%
7	**23403/14408/3037/241**/20268	25-Jan-2020	Australia	1952	3%
8	**23403/14408/3037/241**/29836	26-Feb-2020	UK	1781	2%
9	**11083/14805/26144**/2558/2480	9-Feb-2020	UK	1419	2%
10	/	dic-20	China	1380	2%
11	***28144/8782***	26-Dec-2019	China	1152	2%
12	**23403/14408/3037/241**/25563/18877	21-Feb-2020	Lebanon	1115	1%
13	**23403/14408/3037/241**/25563/29836/1059	21-Feb-2020	France	1103	1%
14	**11083/14805/26144**/17247	25-Feb-2020	UK	1073	1%
15	**23403/14408/3037/241**/15324	3-Mar-2020	Switzerland	1045	1%
16	**23403/14408/3037/241**/25429	7-Mar-2020	UK	1035	1%
17	**23403/14408/3037/241**/25563/1059/27964	8-Mar-2020	USA	1022	1%
18	**23403/14408/3037/241**/28881/28882/28883/313	27-Feb-2020	Switzerland	1021	1%
19	**23403/14408/3037/241**/28881/28882/28883/23731/10097	2-Mar-2020	Denmark	893	1%
20	**1440**/2891	23-Feb-2020	UK	891	1%

Geographical distribution of haplotypes 1,2 and 3 are described in the **S6 Fig**. Haplotype 1 (241, 3037, 14408, 28881, 28882, 28883) is more common in South America and Europe, haplotype 2 (23403, 14408, 3037, 241, 25563, 1059) has mainly spread to North America and haplotype 3 (241, 3037, 14408, 23403) to Africa and Europe. Haplotype 3 is present in 48% of Italian sequences, and haplotype 1 in 36%.

At the beginning of 2021, a new viral strain characterized by 23 variants (6 synonymous and 17 non-synonymous) and associated with a high diffusion capacity was reported in the United Kingdom [24]. One of these mutations, the p.Asn501Tyr mutation located at the RBD of the S protein, has been previously reported to be an adaptive mutation in a mouse model subjected to serial passages of a human SARS-CoV-2 by means of intranasal inoculations [25]. This mutation seems to favor virus binding to the human ACE2 receptor, thus leading to a phenotype of increased virulence in mice [23]. However, p.Asn501Tyr variant and other 8 of the 17 variants (3267C>T p.Thr1001Ile, 6954T>C p.Ile2230Thr in the *ORF1ab* gene; 23063A>T, 23271 C>A p.Ala570Asp, 23604C>A p.Pro681His, 23709C>T p.Thr716Ile in the *S* gene; 27972C>T p.Gln27*, 28048G>T p.Arg52Ile in the *Orf8* gene; 28977C>T p.Ser235Phe in the *N* gene) were observed only in a few sequences in our dataset, but never in linkage with each other, suggesting a synergistic effect of the variants in this new viral strain (**S3 Table**). In particular p.Asn501Tyr was observed in 2 sequences at the beginning of April, one in Brazil and the other in USA. Later, starting from June 2020, the same mutation was identified in 34 Australian sequences.

In conclusion, our analysis emphasizes that the most frequent SARS-CoV-2 variants in early phases of COVID-19 pandemic clustered into four major clades, characterized by different geographical localization, likely reflecting a temporal and spatial spread of virus strains. Moreover, we determined that the most frequent variant, the missense variant p.Asp614Gly in the S protein, is in strong linkage with three additional variants, suggesting a potential functional cooperation in providing a selective advantage to the virus.

Despite the limitations of this study, as the fact that most sequences in the first year of COVID-19 pandemic were reported by UK, and the unavailability of clinical data to correlate the different variants to disease outcome, our analysis provides a portrait of SARS-CoV-2 temporal and spatial spread during the first phases of the pandemic.

During last year several novel virus variants have been sequenced, in particular the World Health Organization (WHO) identified variants of concern (VOCs), which cause an improved virulence and transmissibility or affect the efficacy of diagnosis, treatment or vaccines [26]. The most recently described VOC, the so called Omicron, has been first documented in South Africa in November 2021, where it has been responsible for the forth waive of COVID-19. This variant has raised concerns about its rapid spread and potential escape to vaccines. According to still preliminary data however, Omicron seems to have improved transmissibility but decreased severity [27]. Further studies are needed to verify whether vaccines retain their efficacy also against this variant.

Considering the continuous circulation of the virus and the emerging of new SARS-CoV-2 variants, it is extremely relevant to associate the different haplotypes to a specific outcome of the disease and to understand whether or not the acquired mutations have functional consequences in terms of infectiveness, clinical severity and potential responsiveness to specific treatments and to vaccines.

## Conclusions

By performing a large scale SARS-CoV-2 analysis of reported sequences in the first year of Covid-19 pandemic, we described the existence of 26538 genetic variants, the most frequent clustering into four major clades characterized by a specific geographical distribution. We also found that the most frequent variant, the missense p.Asp614Gly in the S protein, occurs almost exclusively in concomitance with three other variants, suggesting a high linkage and a potential cooperative effect on the virus fitness.

## Supporting information

S1 Fig Worldwide distribution of available genomic sequences. The number and the percentage of sequences reported in each continent and country are indicated in panel (**a)** and (**b)**, respectively. Only countries with more than 200 sequences are shown.
(PDF)

S2 Fig Geographical distribution of the collection dates of the first sequenced sample in each country. (PDF)

S3 Fig Mutational spectrum of SARS-CoV-2 genome. (**a**) Type of variants are listed with the corresponding number and frequency. (**b**) Variant number distribution. The bar graph shows the amount of the reported sequences with the same number of variants. (**c**) Frequency of variants with a direct effect on the virus’ proteins (missense, nonsense and insertion/deletion). Variants are listed with the corresponding number and frequency of reported mutations.
(PDF)

S4 Fig Frequency and geographical distribution of clade 1. (PDF)

S5 Fig Frequency and geographical distribution of clade1-derived haplotypes 1–3. (PDF)

S6 Fig Frequency and geographical distribution of clade2. (PDF)

S1 Table List of all analyzed sequences with collection dates and presence of most frequents variants. (XLSX)

S2 Table Haplotype identified starting from the 53 most frequent variants. (XLSX)

S3 Table Frequency of each variant in all countries. (XLSX)

S4 Table List of all identified variants. (XLSX)
