# Supplementary figures and images for: Worldwide SARS-CoV-2 haplotype distribution in early pandemic
Source: PLoS One. 2022 Feb 16;17(2):e0263705. doi: 10.1371/journal.pone.0263705 (PMC8849502; doi:10.1371/journal.pone.0263705)

a

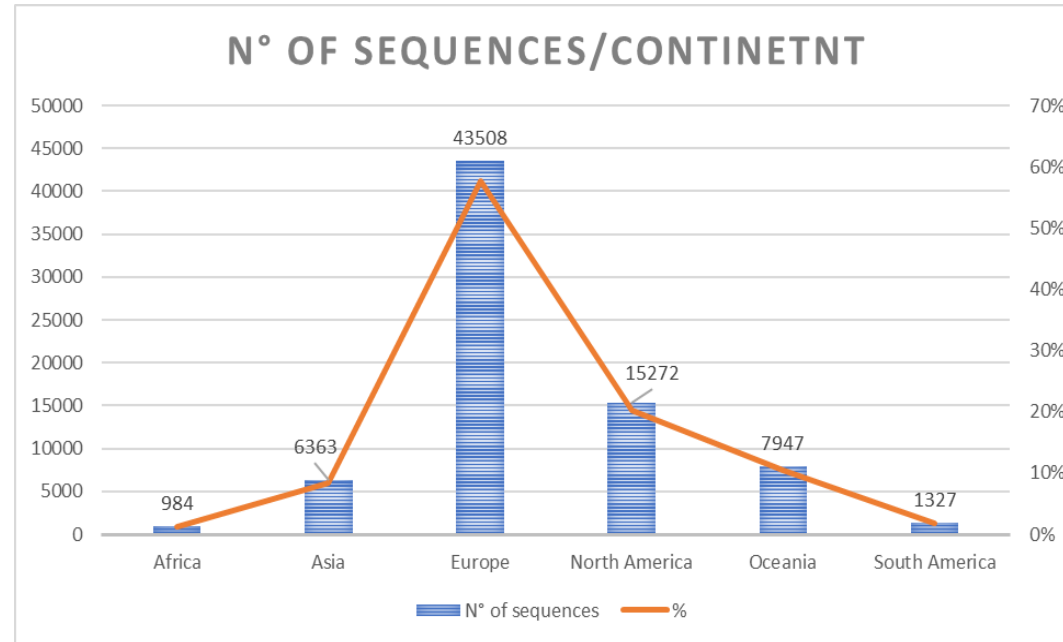

b

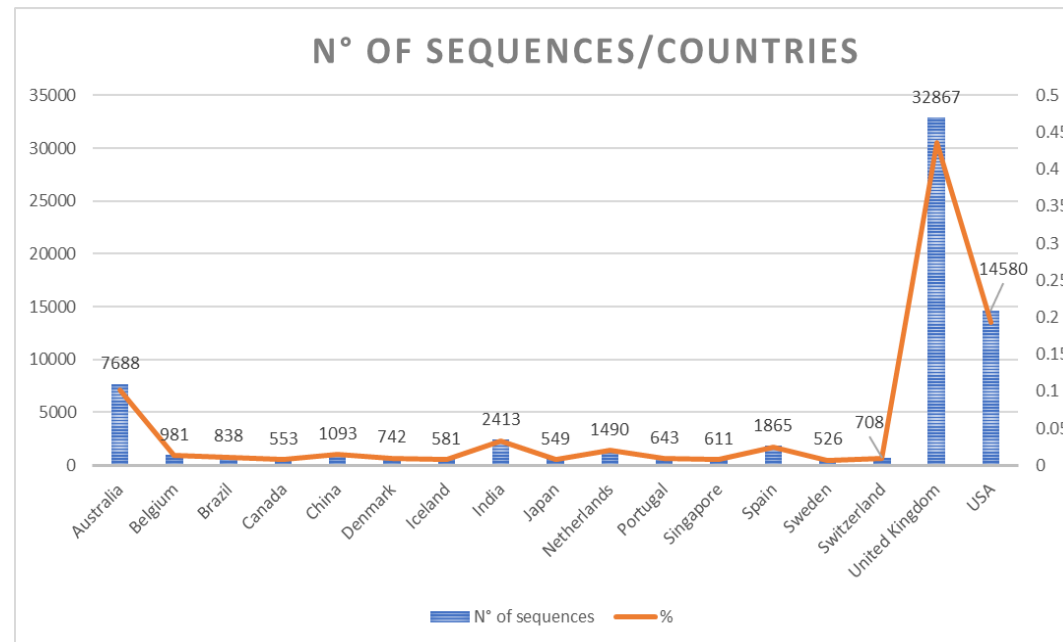

Figure S1

Supplement: S1 Fig — The number and the percentage of sequences reported in each continent and country are indicated in panel (a) and (b), respectively. Only countries with more than 200 sequences are shown. (PDF) [file pone.0263705.s001.pdf]

5

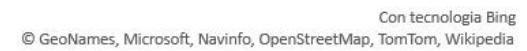

Supplement: S2 Fig — (PDF) [file pone.0263705.s002.pdf]

a

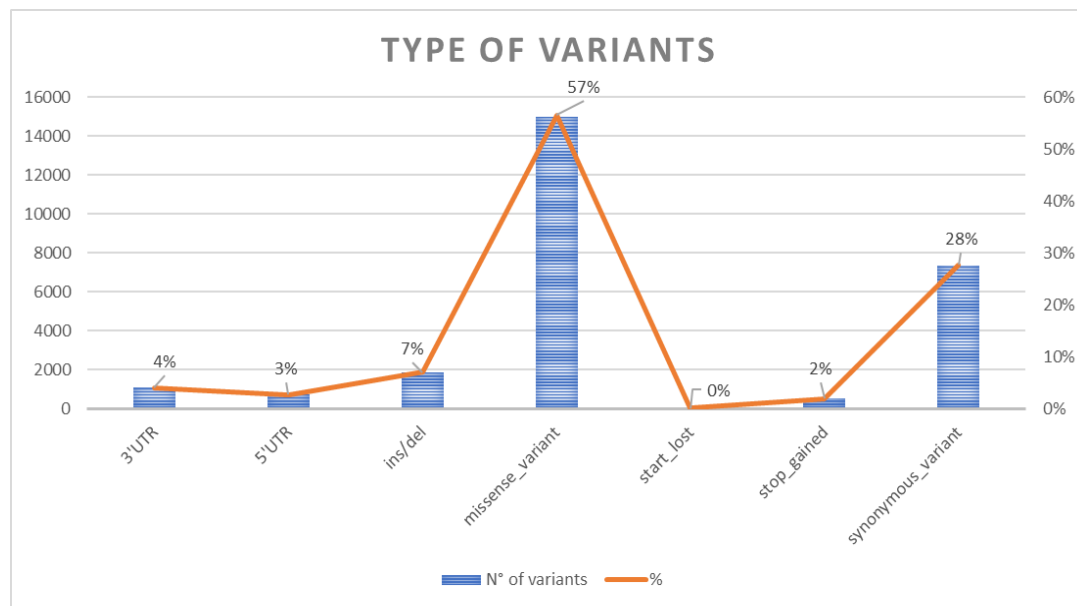

b

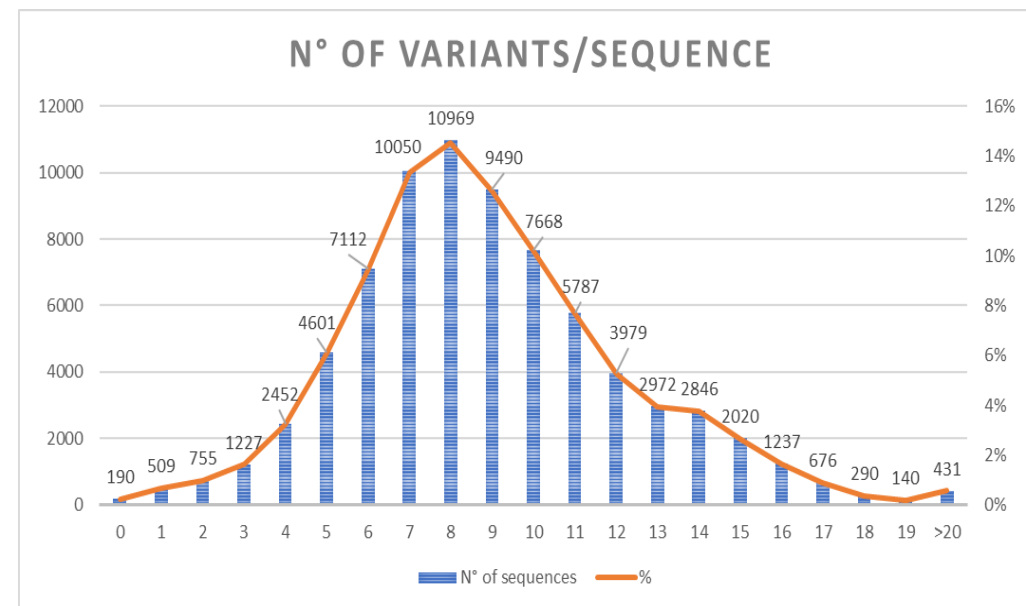

c

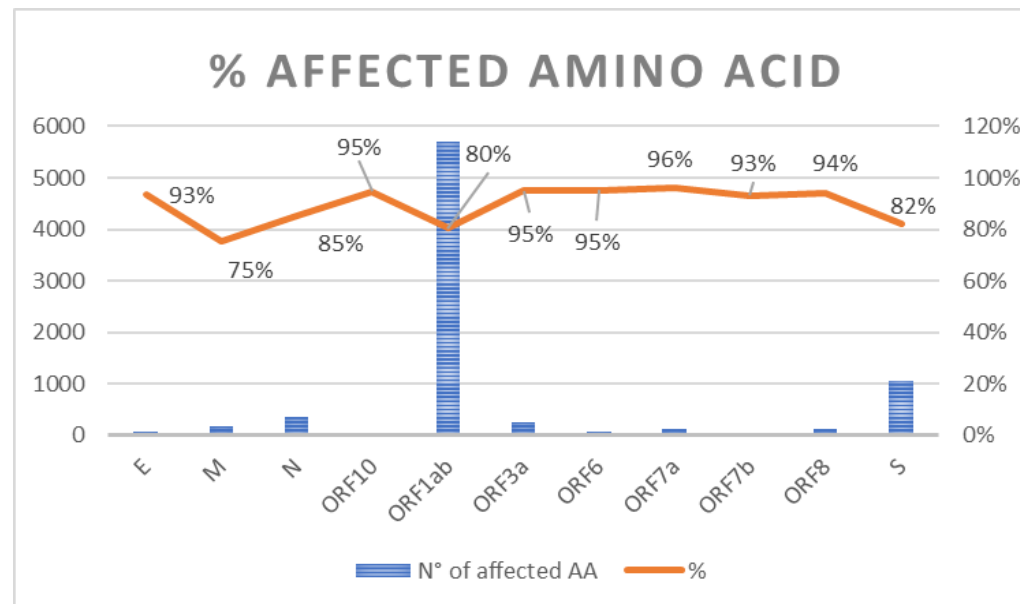

Figure S3

Supplement: S3 Fig — (a) Type of variants are listed with the corresponding number and frequency. (b) Variant number distribution. The bar graph shows the amount of the reported sequences with the same number of variants. (c) Frequency of variants with a direct effect on the virus’ proteins (missense, nonsense and insertion/deletion). Variants are listed with the corresponding number and frequency of reported mutations. (PDF) [file pone.0263705.s003.pdf]

FREQUENCY OF «CLADE\_1» (241, 3037, 14408, 23403)

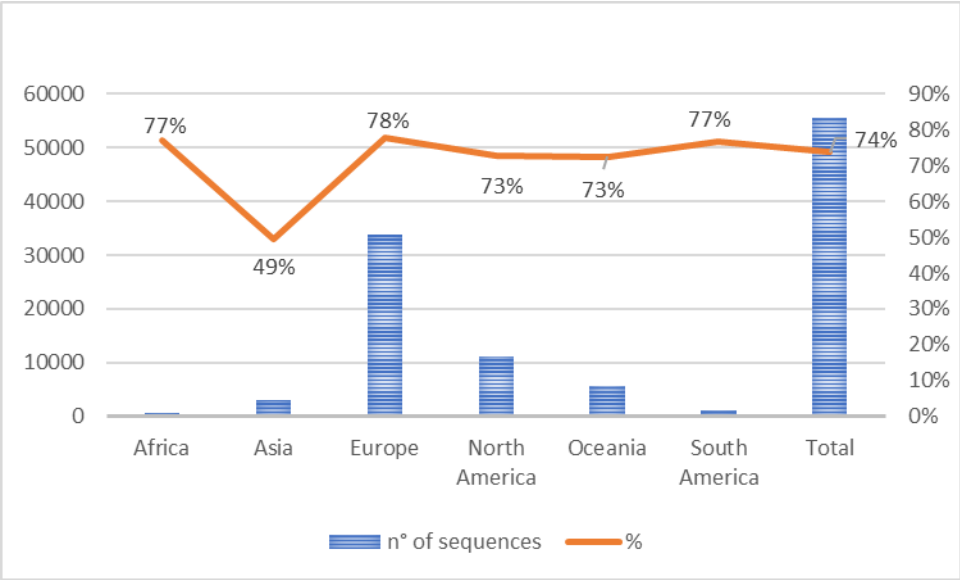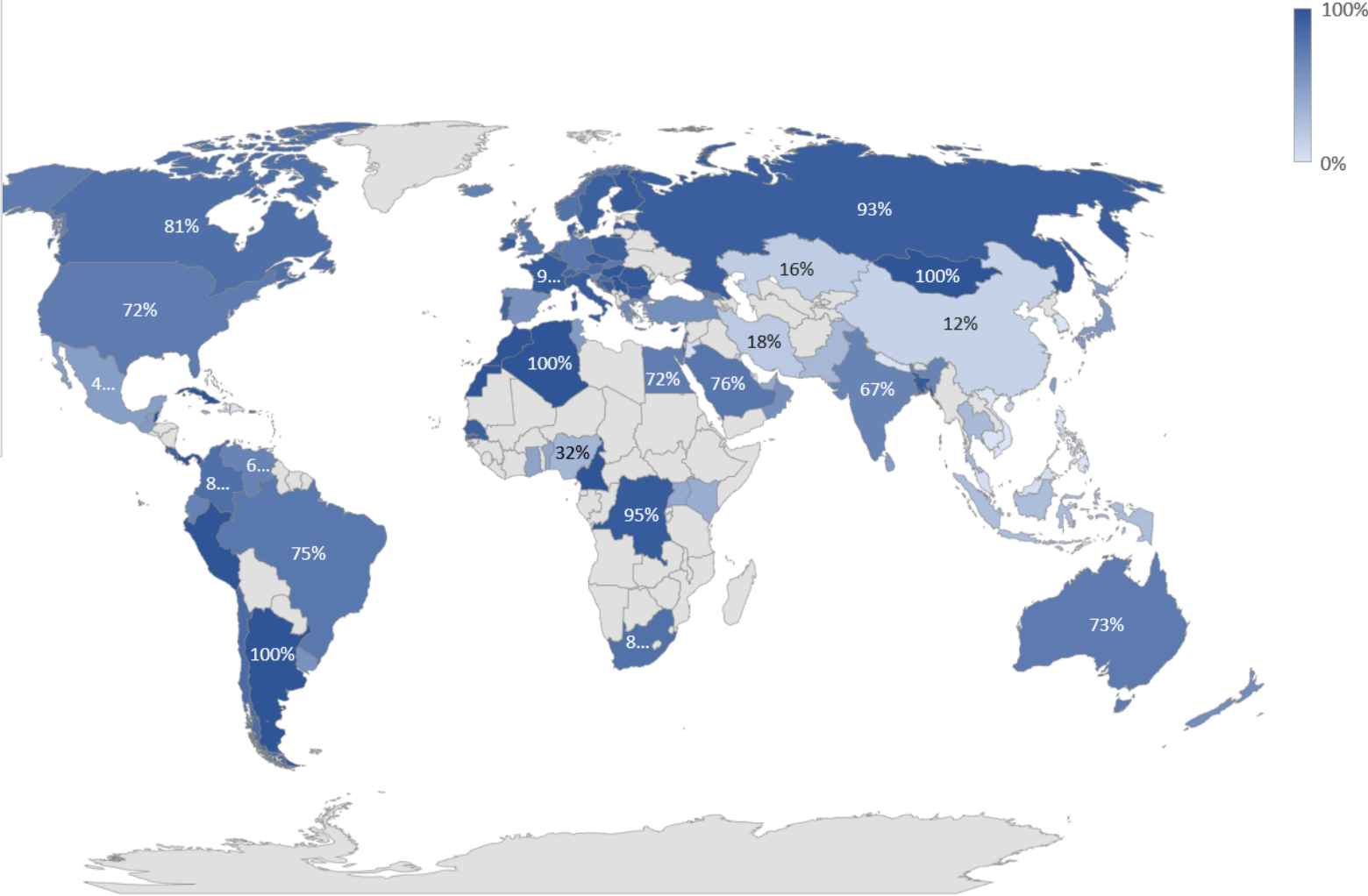

Figure S4

Supplement: S4 Fig — (PDF) [file pone.0263705.s004.pdf]

## FREQUENCY OF «CLADE\_2» (8782,28144)

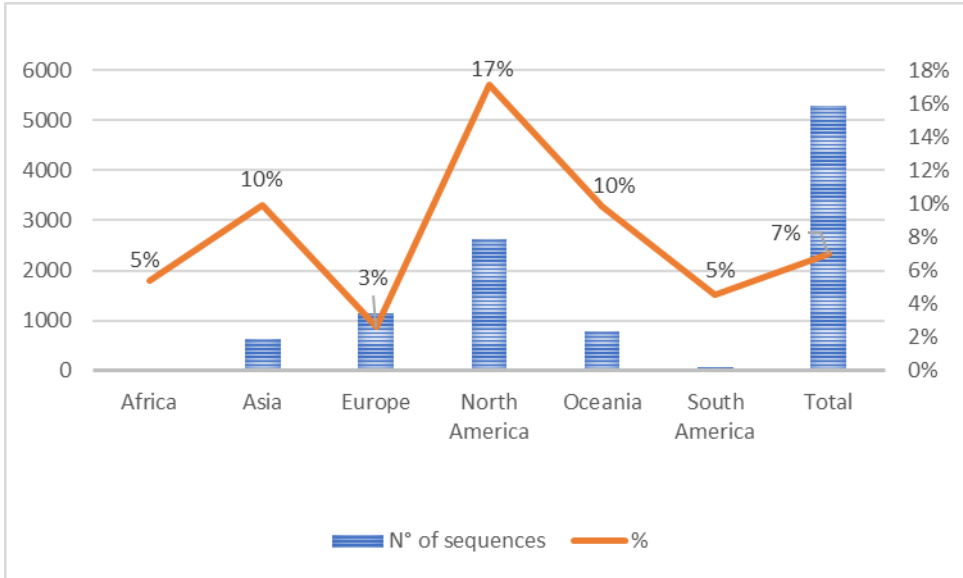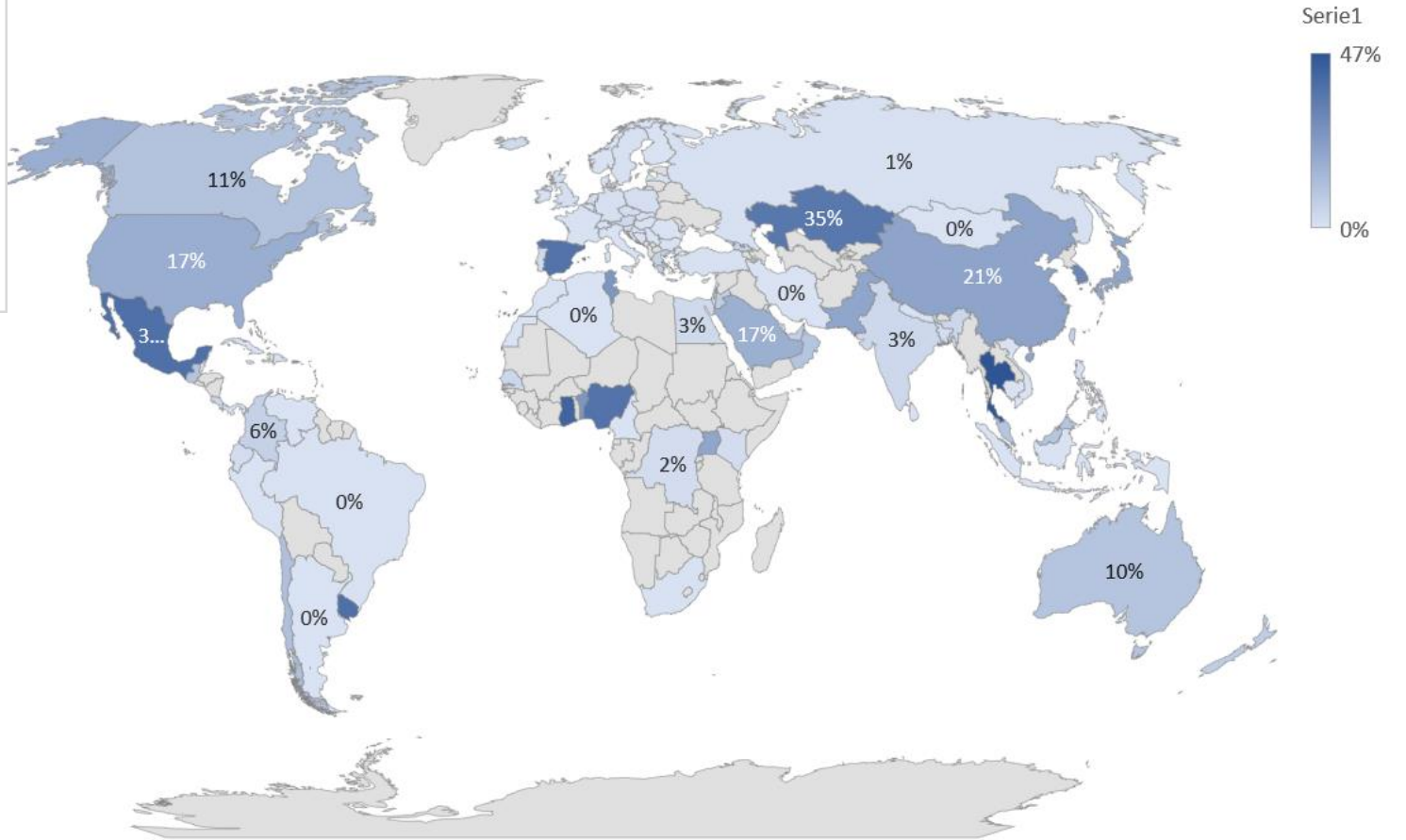

Figure S5

Supplement: S5 Fig — (PDF) [file pone.0263705.s005.pdf]

## DISTRIBUTION OF HAPLOTYPES 1, 2, 3

### HAPLOTYPE 1

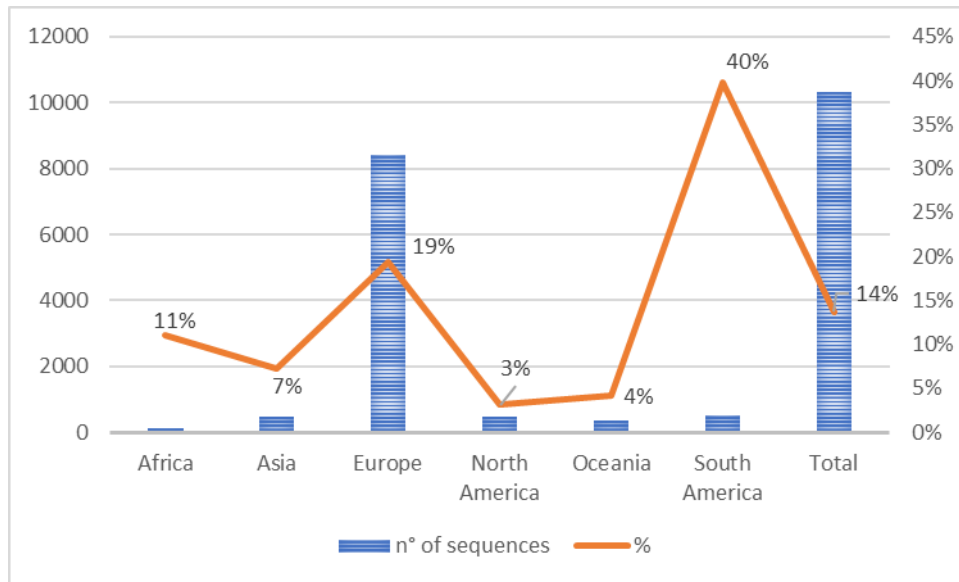

### HAPLOTYPE 3

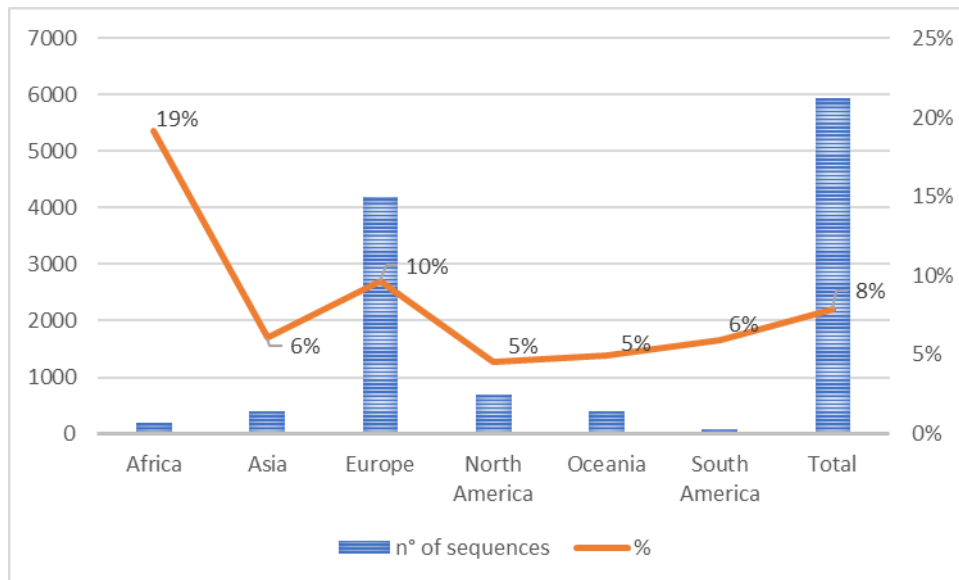

### HAPLOTYPE 2

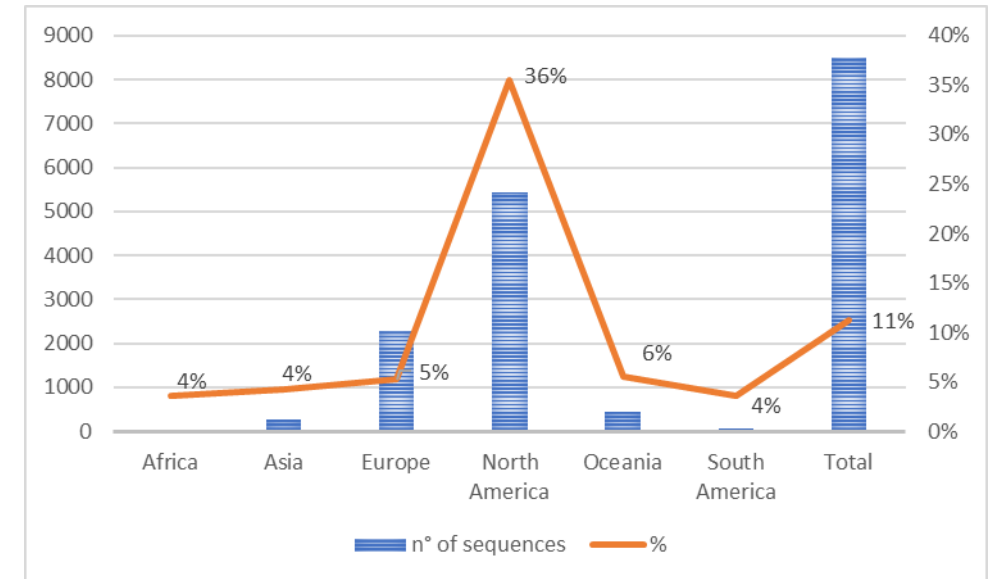

Figure S6

Supplement: S6 Fig — (PDF) [file pone.0263705.s006.pdf]
